# Chondromyxoid fibroma of rib with a novel chromosomal translocation: a report of four additional cases at unusual sites

**DOI:** 10.1186/1746-1596-2-44

**Published:** 2007-11-24

**Authors:** Henry B Armah, Richard L McGough, Mark A Goodman, Susanne M Gollin, Urvashi Surti, Anil V Parwani, Uma NM Rao

**Affiliations:** 1 Department of Pathology, Presbyterian-Shadyside Hospital, University of Pittsburgh Medical Center, Pittsburgh, PA, USA; 2 Department of Orthopedic Surgery, Presbyterian-Shadyside Hospital, University of Pittsburgh Medical Center, Pittsburgh, PA, USA; 3 Pittsburgh Cytogenetics Laboratory, Magee-Womens Hospital, University of Pittsburgh Medical Center, Pittsburgh, PA, USA

## Abstract

**Background:**

Chondromyxoid fibromas (CMFs) are rare benign chondroid/myxoid matrix-producing tumors that occur in metaphyses of long tubular bones, and very rarely in small bones of hands and feet. Flat bone involvement is even more uncommon. Prior cytogenetic analyses have identified complex abnormalities involving chromosome 6 in the majority of cases.

**Methods:**

A search for CMF over an 8-year period (1999–2006) from the surgical pathology files of our institution yielded 16 cases. Four cases occurred in relatively unusual regions, three from the small bones of distal extremities and one from the rib. The rib lesion wassubmitted forroutinecytogenetic analysis.

**Results:**

Radiographic studies revealed that all four lesions were well-defined expansile radiolucent lesions which expanded the bony cortices with lobulated margins, sclerotic rim, septation, and no calcification. Morphologically, all four lesions showed typical features of CMF and had low proliferative index with Ki-67. Cytogenetic analysis on the rib lesion revealed a novel chromosomal translocation, t(1;5)(p13;p13). None of the four patients had a recurrence after a mean duration of follow-up of 24 months.

**Conclusion:**

CMF originating in unusual locations should be distinguished from chondrosarcomas, especially on small biopsies, and should be included in the differential diagnosis. As previously noted in the literature, the cells can be positive for actin but unlike conventional chondroid neoplasms can be negative for S-100. To our knowledge, this is the first report describing a novel chromosomal translocation, t(1;5)(p13;p13) in CMF.

## Background

Chondromyxoid fibroma (CMF) is a rare, peculiar, and benign primary bone tumor that show heterogeneous cytomorphology with a spectrum of chondroid, fibroblastic, and myxoid areas. CMF was originally described and distinguished from other aggressive bone tumors by Jaffe and Lichtenstein in 1948 [1]. It usually presents in the second to third decade, has a male to female ratio of 2 to 1, and is found most often in the metaphysis around the knee in the proximal tibia, proximal fibula or distal femur [2-9]. CMF is thought to be one of the least common bone tumors, representing less than 1% of all primary bone neoplasms and less than 2% of benign bone neoplasms [2,4,10,11]. It occurs most frequently in young adults during the second or third decades of life and is more frequent in males than females [2-9]. Although CMF may involve any bone of the skeleton, it is most commonly found in the metaphyses of major long tubular bones of the lower extremities, particularly around the knee joint [2,4,7-9,12]. CMF has been described as occurring in the apophyses or intracortically [13-17]. Approximately 25% of lesions occur in flat bones, such as the ilium, metatarsals, vertebrae, skull, facial bones, and ribs [2,4,7-9,12]. Lesions in the ribs are extremely rare with only nine reported cases in the English language medical literature [7,18-22]. Clinically, CMF is slow growing and are commonly asymptomatic lesions that are discovered incidentally on radiography [9]. When symptomatic, their clinical presentation is usually chronic local pain (85%), swelling (65%), restriction of motion and, more rarely, pathological fracture [8,11]. The previously described typical radiological features of long bone CMF is a well-defined metaphyseal lytic lesion with a sclerotic rim, septation, and no calcification [23,24]. Cortical expansion and focal destruction may be observed sometimes, but generally the appearances are of a benign lesion. Calcification and intralesional opacities are uncommon and occurs in about 2% of lesions [23,24].

The classic histological features of CMF are lobules of spindle-shaped or stellate cells with abundant myxoid or chondroid intercellular material separated by zones of more cellular tissue rich in spindle-shaped or round cells with a varying number of multinucleated giant cells of different sizes [4,7-10]. Mitoses are exceptionally rare in CMF [4,7-10]. Although it has been suggested that enlarged, pleomorphic nuclei and a prominent mucinous or myxoid component may indicate a higher recurrence rate, histological features are probably not predictive of recurrence [4,7-10]. Recurrence may be more frequent in younger patients [4,8-10]. The identified predisposing factors accounting for the recurrence of CMF are its friable nature resulting in incomplete removal [7,24]. Despite occasional nuclear atypia and local recurrences, CMF is a benign neoplasm and there have been no reports of metastases. Curettage and concurrent bone grafting with allograft bone or polymethylmethacrylate (PMMA) resulted in a much lower recurrence rate than curettage alone, whereas tumors treated by excision did not recur [4,7-10,24]. Local recurrence of CMF following surgery is well recognized and occurs in 3–22% of cases [7-10,25]. Malignant transformation of CMF is a rare event and is reported to be 1–2% [8,25]. Two cases of malignant transformation of CMF after irradiation have been reported [8,9]. Adequate surgical ablation is curative.

The precise histogenesis of CMF is uncertain and is a matter of continuing speculation. A cartilaginous origin, originally proposed on morphological grounds, was subsequently supported by ultrastructural studies and the demonstration of S-100 protein by immunohistochemical studies [5,26-28]. Neilsen [27] showed myofibroblastic, myochondroblastic, and chondrocytic differentiation in CMF on the basis of variable immunostaining for smooth muscle actin (SMA) and S-100 protein. Therefore, it has been suggested on the basis of immunohistochemical studies that CMF resembles chondroblastoma, particularly in relation to the finding of S-100 immunoreactivity; however other types of tumors have similar findings [5,26-28]. Additionally, osteocalcin is present in greater than 50% of CMFs, and in other bone and cartilage tumors, especially chondroblastomas [5].

Karyotypes of fifteen cases of CMF are reported in the English language medical literature, with twelve cases reporting non-random clonal abnormalities involving chromosome 6 [29-38]. In the present report, we describe the clinical, cytological, histological, immunohistochemical and cytogenetic features of four cases of CMF that arose in the rib, finger and foot.

## Methods

Sixteen cases of CMF were retrieved from the surgical pathology files of our institution over an 8-year (1999–2006) period. Four of these 16 cases occurred in relatively unusual regions, including one from metatarsal, two from phalanges, and one from the rib. Clinical information including radiological features and follow-up information was obtained and reviewed for these four cases of CMF at unusual sites. Available original histologic and cytologic slides on all four cases were retrieved and reviewed by two of the authors (AVP and UNMR) who concurred on the diagnoses. Immunohistochemical staining for a panel of antibodies (Table 1) was performed on histologic sections of each of the four cases using standard protocols with a 3-step avidin-biotin-streptavidin procedure. A computer tomography (CT)-guided fine needle aspiration (FNA) biopsy of the right anterior second rib mass (case no. 2) was done prior to the subsequent definitive resection, and immunocytochemical staining for S-100 and Ki-67 was performed for this case. For all immunohistochemical and immunocytochemical analyses, appropriate negative and positive controls were included. A fresh unfixed tumor sample of the resected right anterior second rib mass (case no. 2) was submitted to the Pittsburgh Cytogenetics Laboratory in RPMI tissue culture medium for cytogenetic analysis during the intraoperative evaluation for negative resection margins, since a diagnosis of CMF was favored for the prior CT-guided FNA biopsy. The tumor tissue was treated with collagenase to enzymatically dissociate cells, which were then cultured for 7 to 9 days. Metaphase cells were harvested and chromosomes were GTG-banded using standard procedures.

**Table 1:** Antibodies used in the immunohistochemical study of three cases of chondromyxoid fibromas

**Antibody**	**Manufacturer**	**Pretreatment**	**Source; dilution**
S-100	Ventana (Tucson, AZ)	CCI; 30 mins; 37°C	Mouse; prodilution
SMA	Ventana (Tucson, AZ)	CCI; 30 mins; 37°C	Mouse; prodilution
AE1-AE3	Ventana (Tucson, AZ)	Protease 1; 4 mins; 37°C	mAb; prodilution
EMA	DAKO (Carpinteria, CA)	None	mAb; 1:1000
CD34	Ventana (Tucson, AZ)	CCI; 30 mins; 37°C	Mouse; prodilution
Ki-67	Ventana (Tucson, AZ)	CCI; 30 mins; 37°C	Mouse; prodilution

## Results

### Clinical and radiological features

**Figure 1:**
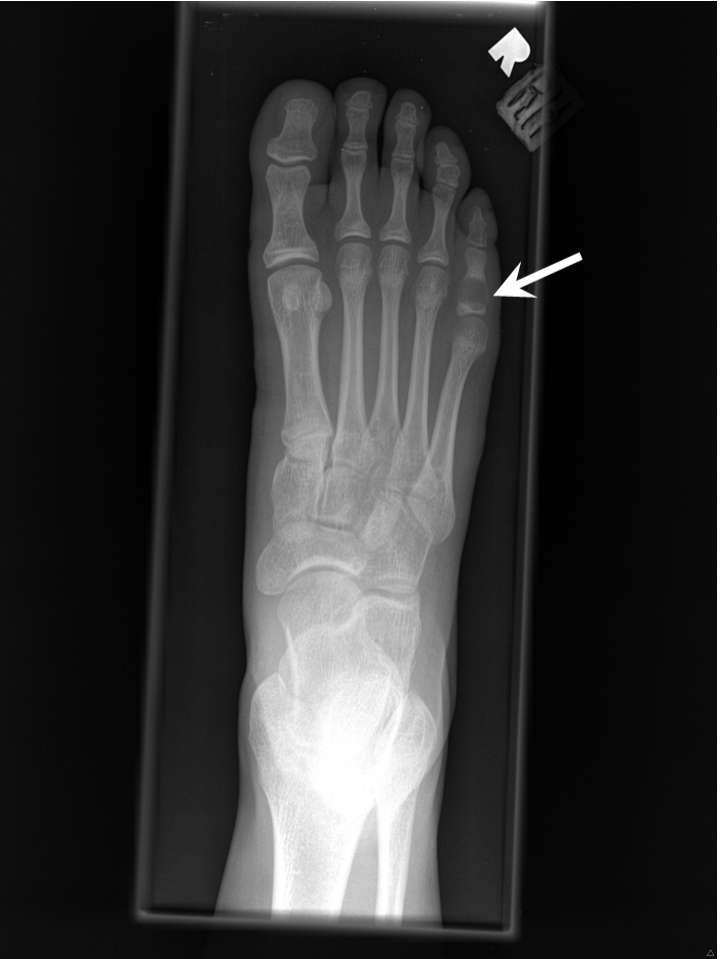
X-ray of the right foot (case no. 1) showing a radiolucent lesion of the proximal metaphysis of the fifth proximal phalanx with scalloped and sclerotic borders, pronounced thinning of the medial and lateral cortices, and without radiographic evidence of an acute fracture, calcification or malignancy (arrow). R indicates the right side.

**Figure 2:**
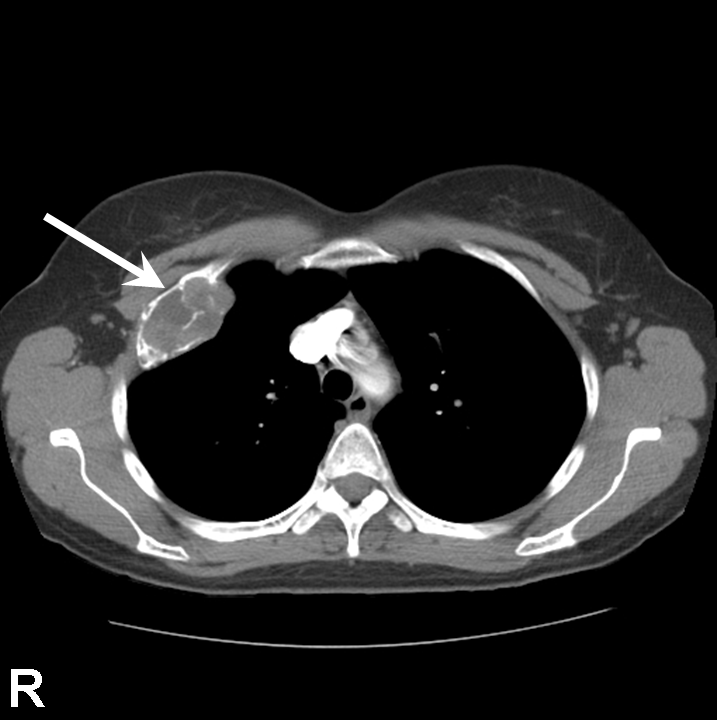
Computed tomography scan of chest (case no. 2) demonstrating a radiolucent lesion of the right anterior second rib with cortical bubbling or blebbing, and without radiographic evidence of an acute fracture, calcification or malignancy (arrow). R indicates right side.

The clinical and radiological features of the four cases of CMF are summarized in Table 2. The patients ranged in age from 14 to 55 years (mean, 32.25 years). Three patients were female and one was male. A right-sided preponderance was observed (3/4 cases). Two cases each presented before and after skeletal maturity. The anatomical distribution of the four lesions was anterior second rib, proximal phalanx of fifth toe, distal phalanx of fourth finger, and second metatarsal. The size of the lesions ranged from 1.3 to 6.1 cm (mean, 2.85 cm). The duration of symptoms prior to diagnosis ranged from 9 to 36 months (mean, 16.75 months). Three cases presented with a painful lump and one with pain only. None of the lesions were found incidentally by radiography. The typical radiological features of a well-defined expansile radiolucent lesion with scalloped or lobulated margins, a sclerotic rim, septation, and no calcification were observed in all four cases of CMF (Figures 1 &2). Three patients with CMF of the foot and hand underwent curettage along with bone grafting using polymethylmethacrylate (PMMA). The patient with CMF of the anterior right second rib underwent a wide "enbloc" resection with chest wall reconstruction using Gore-Tex patch. None of the four patients had a recurrence after a mean duration of follow-up of 24 months.

**Table 2:** Clinical and radiological features of the three cases of chondromyxoid fibromas

**Case no.**	**Age (years)/sex/side**	**Anatomic site/size**	**Clinical presentation/duration**	**Radiologic findings**	**Treatment**		**Follow-up (months)**
						
					**Surgery**	**Bone grafting**	
1	14/F/R	Proximal phalanx of fifth toe/1.3 cm	Painful lump/9 months	Radiolucent lesion with scalloped and sclerotic borders, and no calcification	Curettage	Yes, with PMMA	22, NR
2	43/F/R	Anterior second rib/6.1 cm	Painful lump/3 years	Radiolucent lesion with scalloped and sclerotic borders, and no calcification	Wide enbloc excision with complete resection, and Gore-Tex patch chest wall reconstruction	No	27, NR
3	55/M/L	Second metatarsal/2.5 cm	Pain/1 year	Radiolucent lesion with scalloped and sclerotic borders, and no calcification	Curettage	Yes, with PMMA	39, NR
4	17/F/R	Distal phalanx of fourth finger/1.5 cm	Painful lump/10 months	Radiolucent lesion with scalloped and sclerotic borders, and no calcification	Curettage	Yes, with PMMA	8, NR

F, female; M, male; R, right; L, left; PMMA, polymethylmethacrylate; NR, no recurrence;

### Pathologic features

#### Macroscopic findings

**Figure 3:**
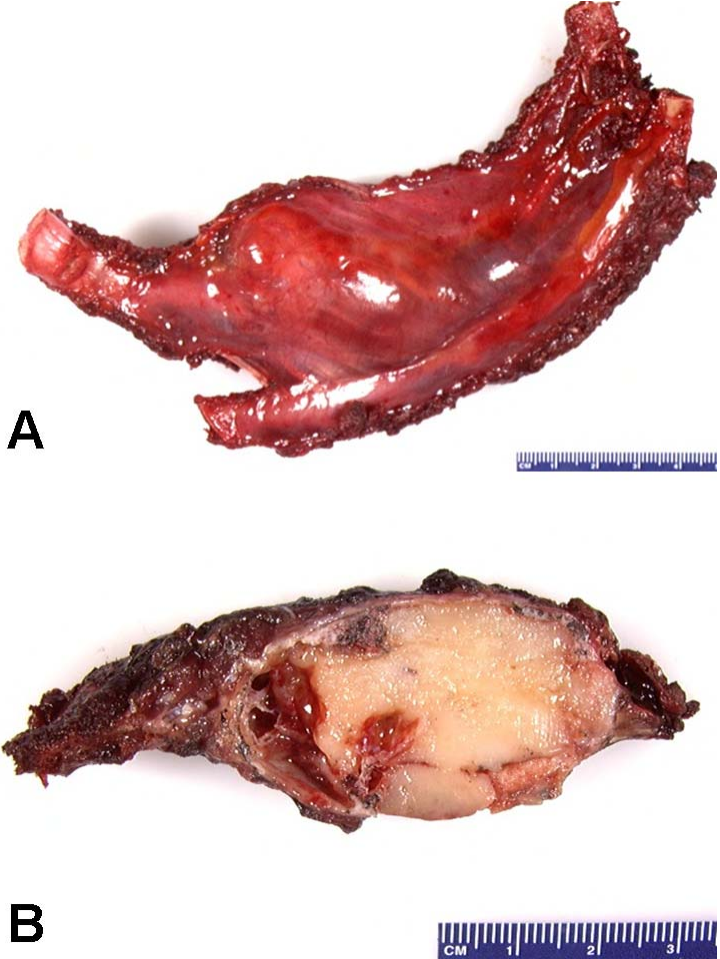
Macroscopic findings of CMF of the resected right anterior second rib (case no. 2). (A) Rib with expansile mass. (B) Cut section of expansile mass showing a distinct glistening, lobulated appearance, with bluish-white color.

The curetted and resected tumor tissue received from all the four cases of CMF were expansile bone masses (Figure 3A) with firm consistency, and their cut sections showed distinct glistening, lobulated appearance, with bluish-white color (Figure 3B).

#### Microscopic findings

##### Cytologic and histologic findings

**Figure 4:**
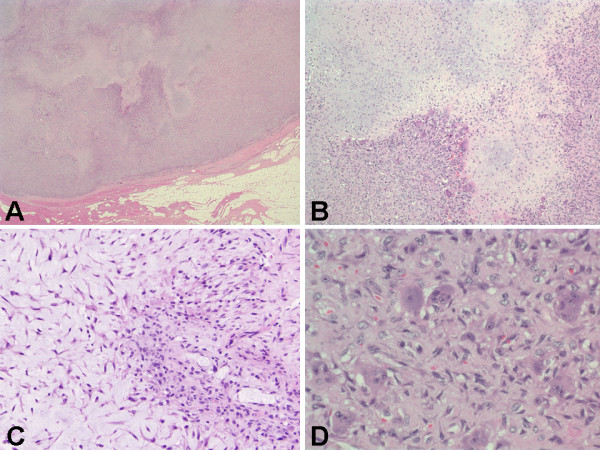
Histologic findings of CMF obtained from resected surgical specimen (case no. 2). (A) Well circumscribed lobules of loose chondroid and myxoid tissue delineated by hypercellular fibrous septae containing bland fibroblasts (H&E, ×4). (B) Characteristic zoned hypercellular areas with osteoclast-like giant cells surrounding chondroid and myxoid areas (H&E, ×10). (C) Chondroid and myxoid matrix containing anastomosing strands of spindle and stellate cells with bland nuclei, finely dispersed chromatin, and inconspicuous nucleoli (H&E, ×20). (D) Hypercellular area containing plump mononuclear cells resembling chondroblasts, and admixed with multinucleated giant cells resembling osteoclasts (H&E, ×40).

CT-guided FNA of the second rib mass (case no. 2) revealed a chondroid neoplasm with low Ki-67 proliferative marker index, and strongly suggested a diagnosis of CMF. The histologic features of all the four resected and curetted tumors were similar to that previously described in the literature. The tumors were composed of lobules of paucicellular pale blue myxoid matrix containing anastomosing strands of spindle and stellate cells with bland nuclei, finely dispersed chromatin, and inconspicuous nucleoli (Figures 4A, 4B &4C). The periphery of the lobules were often rimmed by hypercellular areas that contained similar fusiform to spindle cells mingled with variable numbers of osteocleast-like giant cells (Figure 4D). The nuclei of the stellate cells, especially those in the peripheral areas, showed occasional atypia with enlarged nuclei, coarsely clumped chromatin, and identifiable nucleoli. No mitoses or areas of necrosis were identified. Cells did not occur within lacunae, and no areas of microscopic calcification were found in all four cases of CMF.

##### Immunohistochemical findings

**Figure 5:**
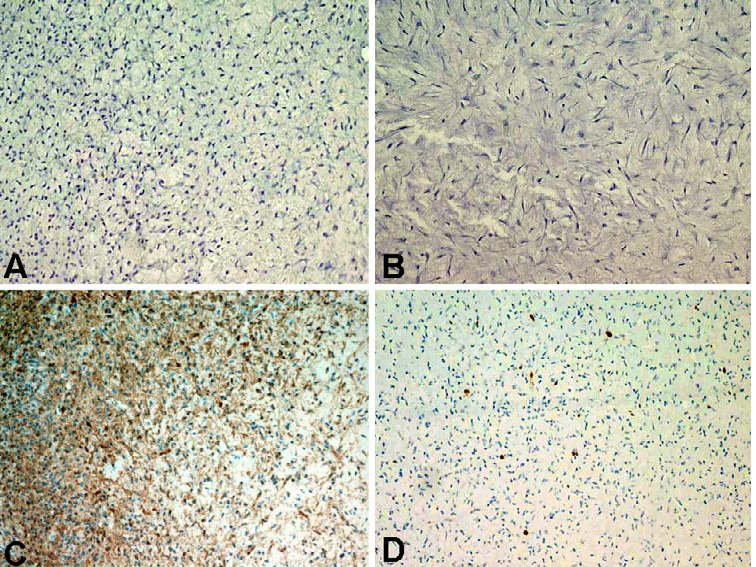
Immunohistochemical findings of CMF obtained curetted surgical specimen (case no. 3). (A) Negative immunostaining with S-100 (Immunoperoxidase, ×20). (B) Negative immunostaining with AE1-AE3 (Immunoperoxidase, ×20). (C) Positive immunostaining of tumor cells with smooth muscle actin (SMA) [Immunoperoxidase, ×20]. (D) Tumor showed low proliferative index with Ki-67 labeling less than 10% of neoplastic cells (Immunoperoxidase, ×20).

The immunohistochemical staining patterns of the four cases of CMF are presented in Table 3. S-100 protein was negative in all four cases of CMF (0%; 0/4) [Figure 5A]. The other immunohistochemical markers that were negative in all four cases of CMF were AE1-AE3 (0%; 0/4) [Figure 5B], epithelial membrane antigen (EMA) [0%; 0/4], and CD34 (0%; 0/4). Smooth muscle actin (SMA) was positive in two of the four cases of CMF (50%; 2/4) [Figure 5C]. All four cases of CMF showed low proliferative index with Ki-67 immunostaining of less than 5% of neoplastic cells [Figure 5D].

**Table 3:** Immunohistochemistry results of the three cases of chondromyxoid fibromas

**Antibody**	**Case no. 1**	**Case no. 2**	**Case no. 3**	**Case no. 4**
S-100	Negative	Negative	Negative	Negative
SMA	Negative	Negative	Positive	Positive
AE1-AE3	Negative	Negative	Negative	Negative
EMA	Negative	Negative	Negative	Negative
CD34	Negative	Negative	Negative	Negative
Ki-67	< 5%	< 5%	< 5%	< 5%

##### Cytogenetic findings

**Figure 6:**
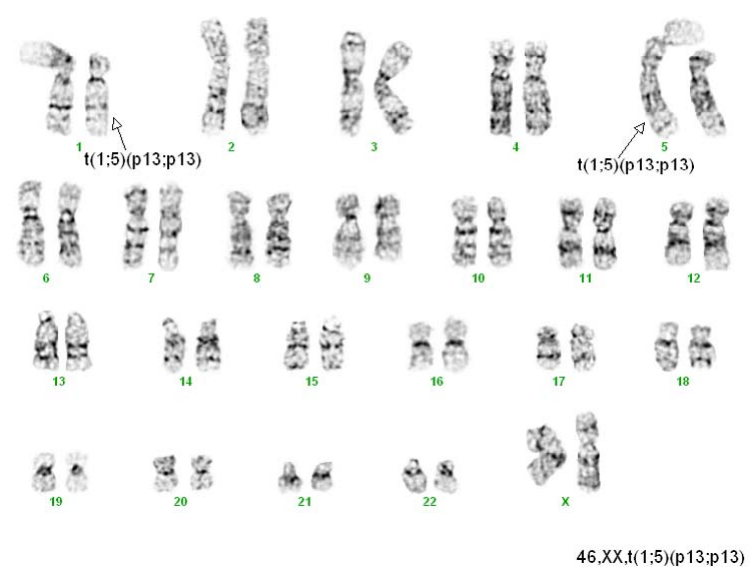
Karyotype of CMF of the right anterior second rib (case no. 2). International System for Human Cytogenetic Nomenclature (ISCN 2005): 46,XX,t(1;5)(p13;p13) [18]/46,XX [3].

Twenty-one trypsin-Giemsa banded metaphase cells were analyzed from 6, 7 and 8-day harvests of monolayer cell cultures derived from the right second rib tumor (case no. 2). Three cells had an apparently normal female chromosome complement. Eighteen cells showed an abnormal pseudodiploid clone with a clonal translocation between the short arms of chromosomes 1 and 5 [46,XX,t(1;5)(p13;p13)] (Figure 6). Four of these eighteen cells had additional non-clonal single cell abnormalities. One cell each had the following chromosome patterns: 46,X,del(X)(p10),t(1;5)(p13;p13); 46,XX,t(1;5)(p13;p13),t(17;19)(q11.2;p11.2); 46,XX,der(1)(1qter→1p13::5p13→5pter),der(5;6)(1pter→1p13::5p13→5q10::6q10→6qter),der(5;6)(p10;q10); and 45,XX,t(1;5)(p13;p13)t(13;17)(q14;p13),-14. The overall karyotype results can be described by the following International System for Human Cytogenetic Nomenclature (ISCN 2005): 46,XX,t(1;5)(p13;p13)[18]/46,XX[3].

## Discussion

The radiological and clinical presentation of all the four cases of CMF in this study is similar to those in the available literature on CMF [3-5,9-11,23-25]. Between 15% and 31% of cases of CMF have been reported to originate in the foot and ankle, and the involvement of the small bones of the feet by CMF has been reported as five times more common than the involvement of the small bones of the hands [2,4,7-9,12]. CMF has no specific radiological distinguishing features. Microscopic calcification is found in about 20% of cases [39]. However, they rarely show calcification on plain X-rays, and are less likely to be confused radiologically with chondroblastomas, enchondromas, or low-grade chondrosarcomas [23,24]. Wilson and colleagues observed that the difficulties with the radiological diagnosis of CMF resulted more from the diversity of sites of involvement and its relative rarity rather than from its radiographic features [23].

CMF rarely arises within the ribs [7,18-22], with only seven cases described so far. The tumor we describe in the rib is histologically typical CMF, with low proliferative Ki-67 index and almost total absence of mitotic activity, but it is unusual not only because of the location and size, but also because of the novel chromosomal translocation found herein and the additional non-clonal random chromosomal aberrations. The latter might be indicative of genetic instability [40] and may reflect on its large size, though this is speculative. There is the need to identify genetic or molecular markers that can aid in confirming the histological impression. Cytogenetic studies of CMF are limited, with 15 previously reported cases in the English language medical literature [29-38]. In the 15 previously reported cases, the tumor was shown to be diploid, with the most common recurring clonal abnormalities involving chromosome 6. In particular, rearrangement of the long arm of chromosome 6 at bands q13 and q25 was most frequent [29-38]. Bridge and Tarkannen both described cases of CMF with aberrations in chromosomes 2 and 5 [29,38], and Ozaki [34] described a case with a gain involving 13q and losses involving 1p, 12q, 16p, 17p, 19p, 19q, 20q, and 22q. The remaining 12 reported cases demonstrated abnormalities in chromosome 6 [30-33,35-37]. Four of these cases demonstrated a pericentric inversion, inv(6)(p25q13) [32,35,36]. One other case demonstrated a different pericentric inversion, inv(6)(p25q23), and the remainder of the cases demonstrated rearrangements in three distinct breakpoint cluster regions, namely 6p23-p25, 6q12-q15, and 6q23-q27 [29-38]. The cytogenetic findings in our case of the rib mass (case no. 2) revealed clonal translocation t(1;5)(p13;p13) and represents the first report of this clonal rearrangement as the sole abnormality in CMF. Although structural chromosomal aberrations involving 1p13 [41-43] and 5p13 [35,44] have been described in chondrosarcomas, these have not been previously reported in CMF.

The morphologic features of CMF may mimic other cartilage-producing neoplasms, especially when occurring in flat bones. Most importantly, CMF can be misdiagnosed as a malignant tumor because of its variable histology, occasional striking cytologic atypia, occasional soft tissue extension, and recurrences. Because of the rarity of CMF, little is known regarding its histogenesis, genetic and biologic characteristics. Based on its variable immunostaining for smooth muscle actin (SMA) and S-100, some investigators have suggested that they are related to chondroblastoma, but CMF is thought to be far less aggressive and considerably different histologically [5,7,9,12,26-28]. The chondroid areas of CMF showed S-100 immunoreactivity in greater than 70% of cases, but did not stain for SMA. Conversely, the peripheral regions surrounding the chondroid areas commonly showed SMA immunoreactivity, but did not stain for S-100 [5,26-28].

The purely histological differential diagnosis of CMF may include chondroblastoma, fibrous dysplasia, chondromyxoma-like or chondroblastic variant of low-grade osteosarcoma, and high-grade myxoid chondrosarcoma [4,7-10]. The distinction is important due to their different natural history, prognosis and management. The location, age of the patient, and the radiological features are extremely important in the diagnosis of majority of bone neoplasms. Chondroid neoplasms of the small bones of distal extremities are seldom malignant, and conversely cartilagenous tumors occurring in the flat bones (including ribs and pelvic bones) are more often malignant than benign. To decrease the risk of diagnostic error, the pathological diagnosis of CMF must be established on the basis of careful correlation of clinical, radiographic and immunohistochemical findings [5,26-28,45]. Chondroblastomas are epiphyseal, unlike CMF which are metaphyseal in location. Additionally, the chondroblasts in chondroblastoma have eosinophilic, polygonal cytoplasm (not stellate as in CMF), and calcification is a conspicuous feature of most chondroblastomas, unlike CMF [5,26-28,45]. Fibrous dysplasia may, at times, show extreme myxoid change; however, the accompanying presence of irregular osteoid seams and the radiological appearance help to make the distinction from CMF [5,26-28,45]. None of the four cases herein reviewed were misdiagnosed as any of the differential diagnosis of CMF mentioned above. Immunohistochemistry is not helpful in distinguishing chondrosarcoma from CMF because both tumors are known to express vimentin and S-100 protein [5,26-28,45]. Chondromyxoma-like or chondroblastic variant of low-grade osteosarcoma, which have very large CMF-like areas, are difficult to differentiate from CMF on a small biopsy. However, mitoses and nuclear atypia, which are seen in osteosarcomas, are not the features of CMF. The diagnostic hallmark of osteosarcoma is the presence of osteoid production, which is totally lacking in CMF [5,26-28,45].

## Conclusion

CMF can and should be distinguished especially from chondromyxoma-like or chondroblastic variant of low-grade osteosarcoma and high-grade myxoid chondrosarcoma, two tumors which are more frequently seen, more aggressive and have the capacity to metastasize. Hence, any lesion labeled as CMF is best confirmed and managed in a specialty bone center, where clinical, radiological and pathological correlation can be optimally achieved. Although often difficult to diagnose, CMFs are relatively easy to treat by curettage with bone grafting or "enbloc" resection, with mininal risk of recurrence. The t(1;5)(p13;p13) is a novel sole clonal cytogenetic abnormality in CMF. Although cytogenetic studies of CMF have not yet yielded a specific causative genetic abnormality, cytogenetic analysis may be of value, and should be performed in all cases of CMF with unusual or borderline clinicopathological features.

## Competing interests

The author(s) declare that they have no competing interests.

## Authors' contributions

**HBA **participated in the histopathological evaluation, performed the literature review, acquired photomicrographs and drafted the manuscript. **AVP**, **RLM**, **MAG **and **UNMR **conceived and designed the study, and revised the manuscript for important intellectual content. **RLM **and **GAM **clinically managed the three cases, and reviewed the clinical and radiological features of the 3 cases.**SMG **and **US **reviewed and interpreted the final cytogenetic diagnosis.**AVP **and **UNMR **reviewed and gave the final histopathological diagnosis. All authors read and approved the final manuscript.
